# How to Turn an Electron Transfer Protein into a Redox Enzyme for Biosensing

**DOI:** 10.3390/molecules26164950

**Published:** 2021-08-16

**Authors:** Antonio Ranieri, Marco Borsari, Stefano Casalini, Giulia Di Rocco, Marco Sola, Carlo Augusto Bortolotti, Gianantonio Battistuzzi

**Affiliations:** 1 Department of Life Sciences, University of Modena and Reggio Emilia, Via Campi 103, 41125 Modena, Italy; antonio.ranieri@unimore.it (A.R.); giulia.dirocco@unimore.it (G.D.R.); marco.sola@unimore.it (M.S.); 2 Department of Chemical and Geological Sciences, University of Modena and Reggio Emilia, Via Campi 103, 41125 Modena, Italy; marco.borsari@unimore.it; 3 Department of Chemical Sciences, University of Padova, Via Marzolo 1, 35131 Padova, Italy; stefano.casalini@unipd.it

**Keywords:** redox biosensing, cytochrome *c*, surface immobilization

## Abstract

Cytochrome *c* is a small globular protein whose main physiological role is to shuttle electrons within the mitochondrial electron transport chain. This protein has been widely investigated, especially as a paradigmatic system for understanding the fundamental aspects of biological electron transfer and protein folding. Nevertheless, cytochrome *c* can also be endowed with a non-native catalytic activity and be immobilized on an electrode surface for the development of third generation biosensors. Here, an overview is offered of the most significant examples of such a functional transformation, carried out by either point mutation(s) or controlled unfolding. The latter can be induced chemically or upon protein immobilization on hydrophobic self-assembled monolayers. We critically discuss the potential held by these systems as core constituents of amperometric biosensors, along with the issues that need to be addressed to optimize their applicability and response.

## 1. Introduction

The heme group is the most abundant metal cofactor in living organisms and is involved in many different biological functions, from O_2_ binding and transport, to redox catalysis, from electron shuttling, to molecular sensing [1,2,3,4]. The reason for this large variety of functions is the chemical versatility of the heme center the reactivity of which is modulated and fine-tuned by the protein matrix, which may control the iron atom axial coordination and spin state, the accessibility to solvent and exogenous molecules, and the polarity of the surrounding environment [3,4,5,6,7].

The multiplicity of physiological roles and the ease of isolation, genetic manipulation, and physico-chemical characterization have made electrode-immobilized native and mutated heme proteins the species of choice for the development of bio(in)organic interfaces for (bio)sensing and catalysis [5,6,8,9,10,11,12,13,14,15]. Exploiting the direct adsorption or covalent attachment of the redox-active protein on the electrode surface proved to be an attractive and promising approach, leading to the development of efficient third-generation amperometric biosensors for substrates of clinical and industrial relevance (O_2_, H_2_O_2_, NO_2_^−^) [5,6,8,9,10,11,12,13,14,15]. Its main drawback consists in the immobilization-induced protein unfolding and inactivation, which may severely hamper the electrochemical and electrocatalytic responses [5,6]. This is particularly relevant for globins, peroxidases, catalases, and cytochrome P450, since their heme *b* is not covalently bound to the protein matrix and can be partially or completely released upon protein denaturation, generating electrochemical responses due to non-native conformers or freely-diffusing heme groups in solution [5,16,17,18]. 

Two approaches are applied to overcome this problem. The first consists in the development of more efficient strategies of protein–electrode immobilization, which effectively couple preservation of the native protein structure with efficient electrical communication [5,6,19,20,21,22,23,24]. The most common immobilization techniques consist in protein physisorption or covalent attachment on (i) the bare electrode surfaces, (ii) electrodes surface-modified with self-assembled monolayers (SAMs) of alkanethiols, or (iii) nanomaterials (Au nanoparticles, carbon nanotubes, graphene), and iv) protein encapsulation into different porous matrices (often incorporating redox mediators or carbon nanotubes) [5,6,19,20,21,22,23,24,25,26,27,28,29,30,31]. 

The second strategy, which is applied in combination with the former, is based on the possibility of effectively modulating and controlling the reactivity of heme proteins by rationally designed mutations in key positions [32,33,34,35,36]. Of particular interest is the replacement of five-coordinated heme *b*-containing proteins (which feature an open heme coordination site for substrate binding) with engineered single and multi-heme cytochromes *c* as the core constituents of the hybrid sensing interfaces [8,9,15,37,38,39,40]. The rationale of this approach is that these species, whose reactivity can be made comparable to that of the replaced proteins, are more robust and more resistant to immobilization-induced unfolding and heme release, due to the presence of covalently bound hemes *c* [8,9,15,37,38,39,40,41,42,43]. The feasibility of this approach has been demonstrated by the ability of mutated cytochromes *c* to catalyze the electrocatalytic reduction of O_2_, H_2_O_2_, and NO_2_^−^ [8,9,15,37,38,39,40,44].

Cytochromes *c* constitute a large and ubiquitous family of redox metalloproteins containing one or more hemes *c* covalently bound to the sidechains of two cysteine residues [45,46,47]. Mitochondrial cytochromes *c* (cytc hereafter) are the most thoroughly characterized species. Cytc is a small globular protein (ca. 13 kDa) that contains a single six-coordinate heme *c* embedded into a hydrophobic environment, and whose axial iron coordination positions are occupied by His18 and Met80 [45,46,47,48]. Under physiological conditions, cytc is located in the intermembrane space of mitochondria, where it participates in the electron transport chain, shuttling electrons from cytochrome *c* reductase (complex III) to cytochrome *c* oxidase (complex IV). 

Due to its relative simplicity, stability, and availability, cytc has long been adopted as a robust model for understanding the molecular details of biological electron transfer and protein folding and stability [41,42,43,45,46,47,48,49,50,51,52,53,54,55,56]. These studies revealed the existence of several non-native conformations, whose 3D structure, heme environment, and axial ligation depend on the solution properties and binding events [41,42,43,45,46,47,48,49,55,56,57,58,59,60,61,62,63,64,65,66,67,68,69,70,71,72,73,74,75,76,77,78,79,80,81,82,83,84,85,86]. This structural flexibility is crucial for controlling the physiological role of the protein, as well as its reactivity in vitro [41,42,43,48,49,51,57,62,63,65,68,75,76,77,78,79,80,83,84,87,88,89,90,91,92,93,94,95,96,97,98,99,100], since the observed changes in heme axial ligation, coordination number, and accessibility confer to cytochrome *c* the ability to bind and catalyze the reduction of small exogenous molecules (such as O_2_, H_2_O_2_ and NO_2_^−^), turning it from an ET protein into an efficient redox enzyme [8,9,15,37,38,39,41,43,44,63,83,84,85,93,98,101,102,103,104,105].

Indeed, it is now well established that cytc is a multi-tasking protein, whose structural properties and biological role depend on external stimuli and cellular location [48,56,57,68,75,93,106]. Its role as an apoptotic trigger is the epitome of this tunable functionality in vivo. This function depends on cytc release into the cytosol, which is activated by binding to the negatively charged glycerophospholipid cardiolipin (CL) found in the inner mitochondrial membrane (IMM) [48,93,106,107]. The interaction with CL induces a conformational transition accompanied by the cleavage of the Fe–S(Met80) bond [48,57,62,63,68,75,77,93,95,103,107,108,109], which imparts cytc with a significant (lipo)peroxidase activity toward CL itself, crucial for the permeabilization of the mitochondrial membrane [48,57,63,68,75,93,107,108,110,111]. Moreover, cytc can move into the nucleus in response to DNA damage, preventing nucleosome assembly and blocking cell survival [48,75,112]; although the mechanism of translocation and the conformations involved in each step are still unknown.

In vitro, chemical denaturation of freely diffusing and surface-immobilized cytc (due to extreme pH values, denaturing agents, organic solvents, or lipid membranes) induces a heme axial ligand swapping similar to that observed upon CL binding in vivo, resulting in non-native low spin His/Lys and His/His axially ligated forms and 5-coordinate high-spin His-ligated species [38,39,44,102,103,113,114,115], featuring good pseudo-peroxidase and nitrite-reductase performances. 

Here, we describe how to turn cytc into an efficient redox enzyme, for the development of bioinorganic interfaces for third generation amperometric biosensors for small substrates (O_2_, H_2_O_2_, NO_2_^−^), through exploitation of (i) point mutations and (ii) chemical- or adsorption-induced unfolding, also in the presence of lipid membranes, coupled with immobilization on the surface of solid electrodes.

## 2. Exploiting Point Mutations

We can identify three major structural features that prevent cytochrome *c* from functioning as an efficient electrocatalyst in its native, folded state (which, on the other hand, are the same that allow it to act as an ET species) [9,44]. The most relevant is the lack of a free position in the iron coordination sphere, in contrast with native heme enzymes, which feature either a five-coordinate iron or a weakly bound sixth axial ligand that is easily displaced by the substrate [1,2]. The second factor is the poor accessibility of the heme pocket to potential substrates. Third, the heme pocket lacks a base catalyst, as the distal His in heme peroxidases, which would facilitate the catalytic mechanism [116].

In the last two decades, the rational design of engineered cytc with non-native enzymatic activity has been devoted to lifting at least one of the aforementioned structural limitations [117,118,119,120,121]. Here, we will focus exclusively on electrode immobilized mutant cytochromes *c* turned into the core components of potential electrochemical biosensors. 

**Figure 1:**
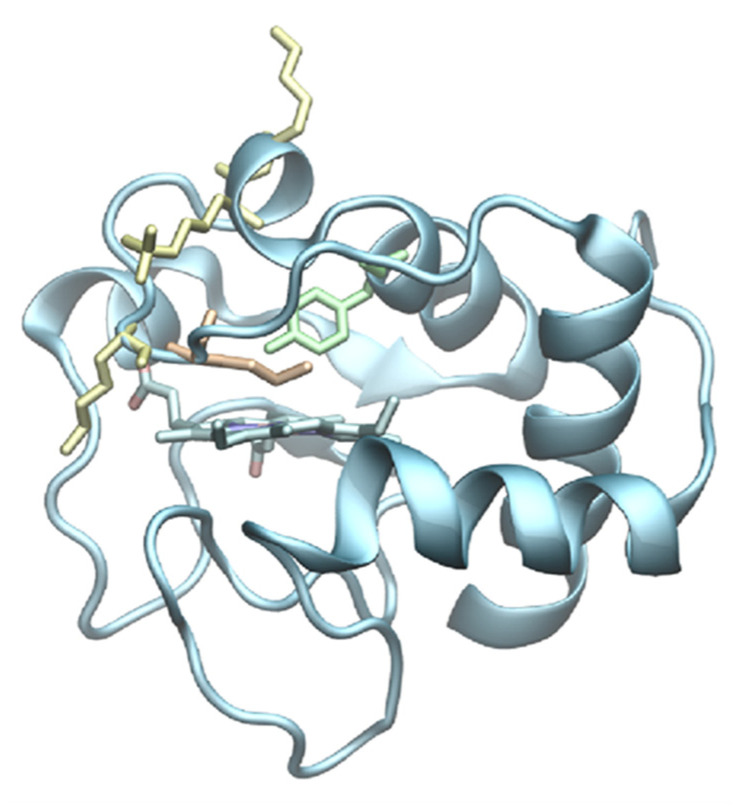
Cartoon representing the 3D structure of wild type yeast cytochrome *c* (PDB 2YCC) highlighting Met80 (orange), Tyr67 (light green), and Lys 72, Lys73, and Lys 79 (yellow).

The most relevant example of an ad hoc designed site-directed mutant of cytochrome *c* endowed with catalytic activity is the M80A mutant, in which the axial iron ligand methionine at position 80 (Figure 1) was replaced by a non-coordinating alanine residue. Despite the mutation, the iron is still hexacoordinated by an OH^−^ ion that protonates with a pK_a_ of 5.6 [37,121,122,123,124,125,126,127] that, however, could easily be replaced by small molecules such as dioxygen, hydrogen peroxide, or anions (e.g., nitrite) [8,9,37,122,123,124,126], as detailed below.

In 2008, our group described the electrocatalytic properties of the M80A mutant of yeast cytc immobilized on self-assembled monolayers (SAMs) formed either by a mixture of mercaptoundecanoic acid/mercaptoundecanol (MUA/MU hereafter) or by 4-mercaptopyridine (4-MP) [37]. On the former SAM, the M80A mutant mainly adsorbs via electrostatic interactions between the negatively-charged SAM headgroups and the surface-exposed lysines of the protein, as indicated by the loss of the redox signals at high ionic strength. Conversely, the M80A mutant is believed to bind to the 4-MP SAM mostly via hydrogen bonds involving pyridine nitrogen and interfacial water molecules. In both cases, the *E°*′ value at 25 °C for the immobilized protein is about 400 mV more negative compared to the wt species under the same conditions, most likely as a consequence of the replacement of the soft S(Met) axial ligand with the hard hydroxide ion [9,37]. 

**Figure 2:**
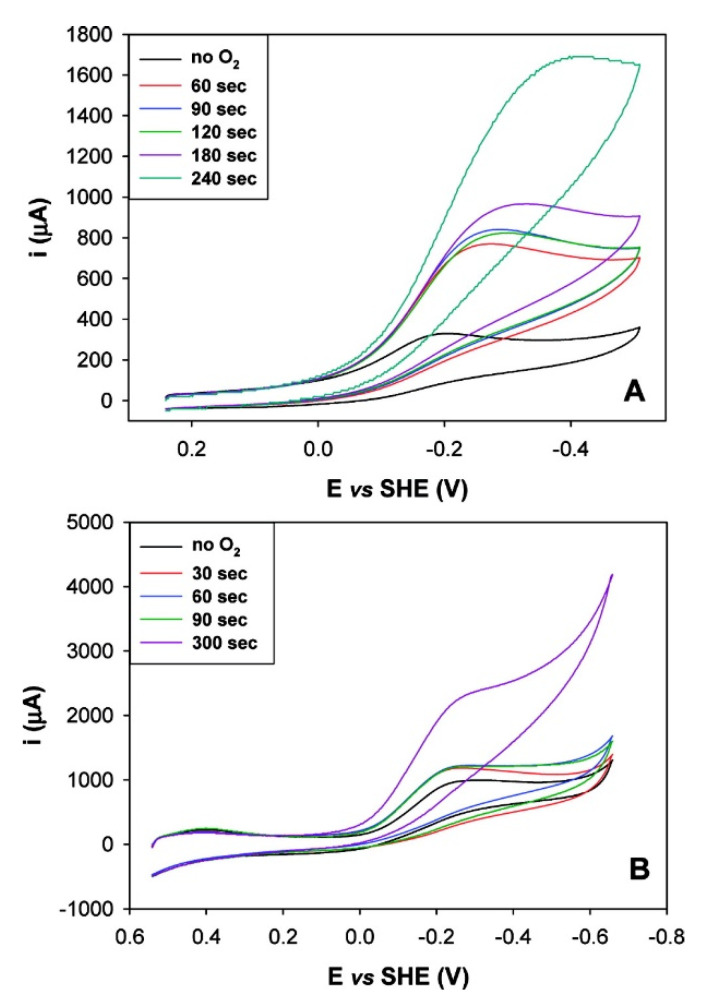
Cyclic voltammograms for the M80A/C102T mutant of yeast iso-1-cytochrome *c* immobilized on a polycrystalline gold electrode coated with a SAM of 1:1 MUA/MU at pH 5.2 (**A**) and 7 (**B**), recorded upon increasing exposure to air. For solution composition and other experimental conditions, the reader is referred to the original literature. Reprinted with permission from ref. [37]. Copyright 2008 American Chemical Society.

For M80A cytc on MUA/MU, electrocatalytic waves were observed upon increasing the concentration of O_2_ in the solution (Figure 2), which could be attributed to the electrocatalytic dioxygen reduction carried out by the adsorbed mutant. Such a response was observed both at slightly acidic (5.1) and neutral pH values [37]. When bound to the 4-MP modified gold electrode, M80A cytc could electrocatalytically reduce O_2_ in a wider pH range (namely pH 5, 7, and 10) [37].

These remarkable electrocatalytic properties are probably due to the large intrinsic affinity of O_2_ for the Fe(II) heme and to the weakening of the bond between the heme iron and the axial ligand (OH^−^ or H_2_O, depending on the pH) upon Fe(III) reduction, which facilitates the binding of O_2_ to the ferrous heme. The most severe limitation to the practical use of the above described construct was the loss of the protein response upon extensive electrochemical cycling, most likely due to oxidative disruption of the MUA/MU or 4-MP monolayers by the radical species yielded by dioxygen reduction (possibly superoxide anion) [37]. 

As stability over prolonged electrochemical cycling is an important prerequisite for amperometric biosensing, the M80A mutation was coupled to two further substitutions to introduce a single, surface exposed cysteine (Cys62) for direct covalent immobilization of the protein on bare Au working electrodes [8]. The M80A/N62C/C102T mutant covalently bound to bare gold was indeed able to electrocatalytically reduce O_2_ with higher catalytic currents over a wider pH range (from 3 to 10) compared to the physiosorbed M80A variant, and, most notably, its electrochemical response did not fade upon prolonged electrocatalytic activity [8]. 

**Figure 3:**
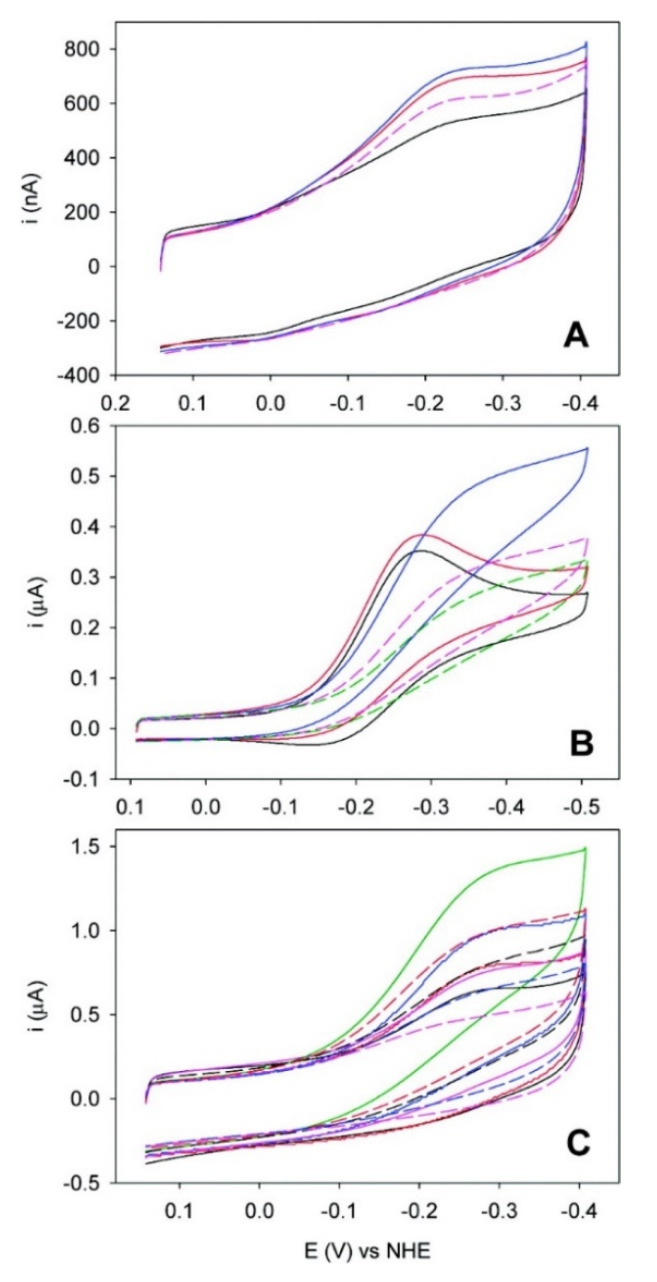
Cyclic voltammograms recorded in the presence of increasing concentrations of sodium nitrite for the M80A variant of yeast iso-1-cytochrome *c* immobilized with different procedures on a polycrystalline gold electrode at pH 7. (**A**) M80A/C102T on a SAM 4-MP (black, no nitrite; red, 10 μM; blue, 20 μM; magenta, 100 μM), (**B**) M80A/C102T on a SAM of MUA/MU (black, no nitrite; red, 1 μM; blue, 5 μM; magenta, 15 μM; green, 20 μM), and (**C**) M80A/N62C/C102T covalently linked to the electrode through an Au-S(Cys) bond (black, no nitrite; red, 1 μM; magenta, 2 μM, blue, 5 μM; green, 10 μM; dashed red, 20 μM; dashed black, 30 μM; dashed magenta, 50 μM). For the solution composition and other experimental conditions, the reader is referred to the original literature. Reprinted with permission from ref. [8]. Copyright 2008 American Chemical Society.

The M80A/N62C/C102T mutant was also able to electrocatalytically reduce the nitrite anion upon immobilization on 4-MP, on MUA/MU, and when covalently bound to bare Au [8]. Independently of the surface confinement strategy, when the ion concentration was increased to the micromolar range, an increase of the cathodic current, accompanied by a decrease of the anodic one, was observed (Figure 3) [8]. 

The proposed two-step mechanism first involves reduction of heme iron (which destabilizes the bond with the OH^−^ anion serving as the sixth ligand) followed by the one-electron reduction of nitrite to NO [8,39,44]:

cytc-Fe(III)-OH^−^ + e^−^ → cytc-Fe(II) + OH^−^ cytc-Fe(II) + NO_2_^−^ + H^+^ → cytc-Fe(III)-OH^−^ + NO (1)

**Figure 4:**
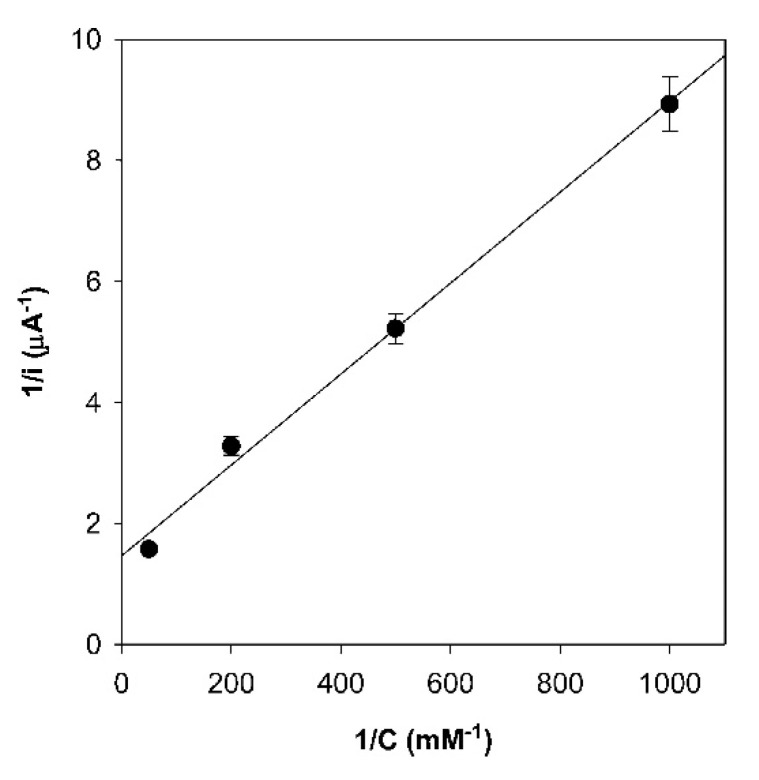
Lineweaver–Burk plot for the electrocatalytic currents yielded by the M80A/N62C/C102T variant of yeast iso-1-cytochrome *c* covalently linked to the electrode through an Au-S(Cys) bond, pH 7, in the presence of increasing nitrite ion concentrations. Reprinted with permission from ref. [8]. Copyright 2008 American Chemical Society.

The catalytic activity of the electrode-immobilized M80A/N62C/C102T mutant was successfully estimated using the Michaelis–Menten model, expressing the Michaelis–Menten equation in terms of current density [8,39,44]:

(2) $$\frac{1}{j_{c}}=\frac{1}{j_{max}}+\frac{K_{M}}{j_{max}\cdot \left[NO_{2}^{-}\right]}$$

where *j_c_* and *j_max_* are the electrocatalytic current density and the maximum current density at substrate saturation, respectively (Figure 4). 

The latter is the electrochemical equivalent of *v_max_* in the usual Michaelis–Menten model and depends on *k_cat_* and on the surface concentration of the catalytically active protein on the electrode. Hence, fluctuations in *j_max_* are directly connected to *k_cat_* changes, provided that the protein coverage of the SAM is conserved. As a consequence, *j_max_* reflects the ability of electrode-immobilized M80A/N62C/C102T mutant to catalyze the reduction of NO_2_^−^ [8,39,44]. *K_M_* indicates the kinetic affinity between the protein and substrate, the smaller the *K_M_* values the higher the protein affinity for NO_2_^−^. 

The cathodic current decreases for [NO_2_^−^] > 20 μM, possibly as a consequence of electrostatic interactions between the anion and the positively charged lysine-rich patches on the protein surface, which might induce a conformational change detrimental to the catalytic activity [8]. The *K_M_* and *j_max_* values are 5.1 μM and 2.14 μA cm^−2^ (Table 1), respectively [8]. *K_M_* values in the micromolar range indicate a significant kinetic affinity of the nitrite ion for the mutant (much larger than that for myoglobin or cytochrome P450, which show larger *K_M_* values, by about three orders of magnitude [128,129]). The *j_max_* values are informative on the catalytic efficiency, which turns out to be comparable to that of a freely diffusing nitrite reductase accepting electrons via heterogeneous ET from surface-immobilized cytc_551_ [130].

Very recently, our group investigated the electrocatalytic properties of a surface-bound M80A mutant in mixed organic/water solvent [82]. Retaining peroxidase-like activity in a non-fully aqueous environment is crucial for exploitation of the construct in real life applications. In particular, M80A was electrostatically physisorbed on a MUA/MU mixed SAM, and its electrocatalytic activity towards H_2_O_2_ reduction was measured in dimethylsulfoxide (DMSO)/water mixtures [82]. The mutant retained its peroxidase-like properties up to 60% *v*/*v* DMSO, while at higher DMSO concentrations a marked decrease of the activity occurred, most likely due to conformational changes, as indicated by changes in the reduction thermodynamics and ET kinetics [82].

Besides substitution of an axial ligand with a non-coordinating residue, the heme pocket was further modified to make it more similar to that of native heme redox enzymes. A key residue in this respect is the highly conserved Tyr67, which plays a crucial structural role, through its extended hydrogen bond network within the heme pocket [127,131,132]. To assess the role played by Tyr67, several research groups produced and characterized cytc variants at position 67, exploring changes at the structural level and in the peroxidase-like activity [117,119,120]. The two most widely explored mutations for yeast and human cytcs were Y67H and Y67R [117,119,120]. Interestingly, the Y67H mutation has little structural effects, despite the weakening of the Fe-S(Met80) bond [120]. On the contrary, in the Y67R mutant of human cytc, the Fe-S(Met80) bond is lost, resulting in an eight-fold increase of peroxidase activity with respect to the wt protein [120], possibly due to the stabilization of the negative charge of the Fe(III)-OOH complex by the newly introduced Arg67 during Compound I formation. 

To assess whether addition of a mutation at Tyr67 to the Met80-to-Ala swapping further enhances the catalytic properties of cytc, our group investigated the electrochemical behavior of the double mutants M80A/Y67H and M80A/Y67A of yeast cytc adsorbed on an MUA/MU-modified gold electrode [9]. The near invariance of the *E°*′ values suggested that the Y67/A-H changes did not induce relevant structural differences compared to the single M80A mutant. Both double variants were able to electrocatalytically reduce H_2_O_2_ in the low micromolar range [9]. An irreversible catalytic activity loss occured at H_2_O_2_ concentrations higher than 5 μM and 18 μM, respectively, which could be ascribed to oxidative damage to the protein and/or loss of the heme iron [9]. 

The catalytic activity of the M80A variant and both double mutants was again estimated using the Michaelis–Menten equation, expressed in terms of current density [9]:

(3) $$\frac{1}{j_{c}}=\frac{1}{j_{max}}+\frac{K_{M}}{j_{max}\cdot \left[H_{2}O_{2}\right]}$$

Perhaps surprisingly, the immobilized M80A/Y67H and M80A/Y67A displayed poorer peroxidase-like capability than M80A, as indicated by lower *j_max_* values (3 and 10 times smaller than M80A for M80A/Y67H and M80A/Y67A, respectively) and slightly higher *K_M_* values (Table 2) [9]. Therefore, despite resulting in a larger heme cavity, the Tyr67 substitution removes a residue that is important for efficient catalytic turnover, most likely thanks to its involvement in the H-bond to hydrogen peroxide or hydroperoxide anion.

A similar approach can be extended to multi-heme proteins. In particular, the M64A and M164A variants of the di-heme cytochrome *c*_4_ from *P. haloplanktis,* in which the Fe-binding methionine of both heme centers is replaced by an OH^−^ ion, have been studied as potential components for third generation biosensors [40]. Indeed, the M64A mutant, thanks to a favorable orientation on a MUA-coated gold electrode, can act as catalyst for dioxygen reduction, while the native C-terminal heme most likely functions as an electron shuttle to the newly introduced catalytic center [40].

## 3. Exploiting Protein Unfolding

Cytochrome *c* unfolds due to acidic pH, denaturing agents (urea and GdCl) or upon interaction with (mainly hydrophobic) surfaces [10,68,76,77,88,108,113,115,127,133,134,135]. In all cases, a remarkable decrease in *E°*′ is observed, due to the replacement of Met80 by a new axial ligand (either an OH^−^ ion or an endogenous His or Lys residue), which selectively stabilizes the ferric protein. 

### 3.1. Chemical Unfolding

Cyclic voltammetric and Surface-enhanced resonance Raman (SERR) spectroscopy studies have shown that in the presence of high urea concentration (>6 M) the *E°*′ of beef heart and yeast cytcs, adsorbed on electrodes surface-modified with MUA/MU and 4-MP SAMs, shifts to approximately 0.4 V more negative values, owing to the replacement of the native Met80 axial ligand by His26 or 33 [101,102,113,115,136]. In similar conditions, the same heme axial ligand swapping is also observed for single, double, and triple mutants of yeast cytc, in which the lysine residues surrounding the solvent-exposed heme edge (Figure 1) are replaced by alanines (K73A, K79A, K72A/K73A, and K72A/K73A/K79A variants) [102,113]. 

In all cases, the adsorbed urea-unfolded proteins catalyze the reduction of O_2_ and H_2_O_2_ [101,102]. The unfolded bis-His ligated conformers, prevailing at large concentrations of unfolding agent [101,102,113,136], are responsible for the observed behavior [101,102], since the native His-Met-ligated form of all immobilized proteins is unable to perform the electrocatalytic reduction of O_2_ and H_2_O_2_. Moreover, the protein layers are stable and can be re-used, as the voltammetric signal of all bis-His-ligated conformers can be restored upon complete removal of dioxygen and H_2_O_2_, and the catalytic currents can be re-obtained upon addition of the substrates [101,102]. Most importantly, the near invariance of the cathodic and anodic peak potentials over the entire range of dioxygen partial pressures and hydrogen peroxide concentrations analyzed indicates that in all cases the catalytic active species is the ferrous form [101,102], which binds and reduces O_2_ and H_2_O_2_, as observed for different heme enzyme-based electrodes [5,6,22,137,138].

The lower affinity of the imidazole ring of histidine for the ferrous heme compared to the sulfur of the native Met80 axial ligand is crucial, because it reduces the stability of the reduced bis-His-ligated conformer, inducing the dissociation of one of the histidine ligands from the iron(II) center [101,102], and thereby making an axial site available for the interaction with O_2_ and H_2_O_2_. On the contrary, the six-coordinate ferrous heme iron of folded wt cytc is not available for substrate binding, due to the strong bond formed with the thioether sulfur of the axial Met ligand.

The observed electrocatalytic activity of the bis-His-ligated cytc conformers towards O_2_ can be accounted for by a mechanism in which O_2_ binds to the ferrous heme upon dissociation of one of the axial His, yielding an oxidative addition that results in a Fe(III)–O_2_^−^ derivative, which then dissociates [101]:

cytc-Fe(III)-His + e^−^ → cytc-Fe(II) − − − His cytc-Fe(II) − − − His + O_2_ → cytc-Fe(III)-O_2_^−^ cytc-Fe(III)-O_2_^−^ → cytc-Fe(III)-His + O_2_^−^ (4)

where cytc-Fe(II) − − − His is the ferrous unfolded yeast cytc, in which one of the metal–His bonds is longer and weakened. Detection of the superoxide ion in the products of the (electro)catalytic reduction confirms the soundness of the above mechanism [101]. 

Likewise, based on literature data showing that in several reactions iron (II) is oxidized to the ferryl group by H_2_O_2_ [139], the following mechanism is proposed for the observed electrocatalytic reduction of H_2_O_2_ mediated by the ferrous bis-His-ligated unfolded conformer [102]:

cytc-Fe(III)-His + e^−^ → cytc-Fe(II) − − − His  cytc-Fe(II) − − − His + H_2_O_2_ → cytFe(IV)=O(His)_uncoordinated_ + H_2_O  cytFe(IV)=O(His)_uncoordinated_ + e^−^ + 2 H^+^ → cytc-Fe(III)-His + H_2_O (5)

Since the *E°*′ of the ferryl group is remarkably more positive than that of the ferric heme, under the conditions of the catalytic event, the former is immediately reduced at the potential of the electrode [102].

The catalytic activity of electrode-immobilized unfolded His–His conformers in 9 M urea was successfully estimated using the Michaelis–Menten model [102]. The data reported in Table 3 show that the urea unfolded His–His conformer of yeast cytc immobilized on both MUA/MU and 4-MP SAMs possesses a large catalytic efficiency for O_2_ reduction [101], which is only marginally affected by the nature of the protein–SAM interactions (electrostatic or through H-bonds). Since cytc unfolding has been associated with human pathologies and disorders involving increased production of partially reduced oxygen species, this result suggests that O_2_ adds to H_2_O_2_ as a substrate for catalytic reactions carried out by denatured cytc, which contributes to the overall oxidative stress of the cell [101].

As for O_2_, the *K_M_* and *j_max_* values for the electrocatalytic reduction of H_2_O_2_ by the immobilized unfolded bis-His-ligated conformers of native cytc and the Lys-to-Ala mutants (Table 2) are indicative of a high catalytic efficiency [102]. In particular, the *K_M_* values are comparable to those of the M80A variant [9], and show that the kinetic affinity of H_2_O_2_ for the heme iron in these proteins is one to three orders of magnitude larger than those for other heme proteins immobilized on surface-modified electrodes [102].

**Figure 5:**
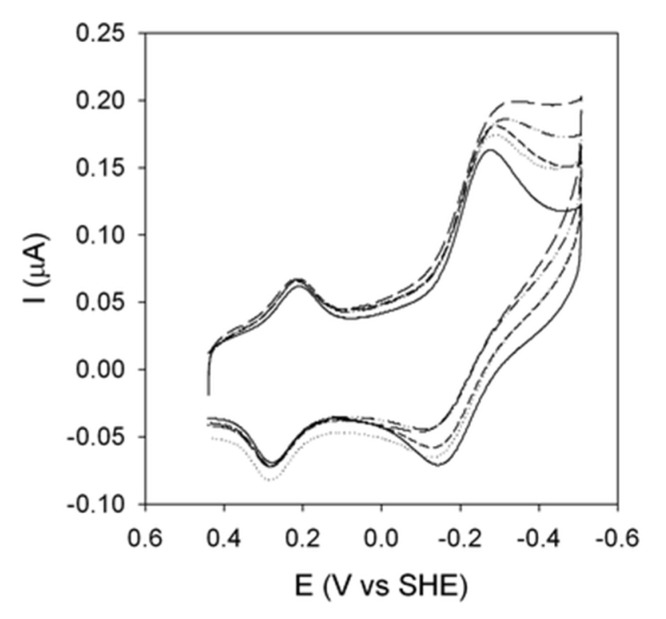
Cyclic voltammograms for a K72A/K73H/K79A mutant of yeast cytochrome *c* adsorbed on polycrystalline gold electrode modified with a SAM of MUA/MU recorded in the presence of increasing concentrations of H_2_O_2_. For the solution composition and other experimental conditions, the reader is referred to the original literature. Reprinted with permission from ref. [15]. Copyright 2012 Royal Society of Chemistry.

Between pH 5 and 7.5, the K72A/K73H/K79A mutant of yeast cytochrome *c* undergoes a reversible, pH-dependent, structural transition, in which the newly introduced His 73 replaces Met80 as the sixth axial heme iron at slightly alkaline pH values [15,38,56,88,140,141]. Upon immobilization on a MUA/MU surface-modified gold electrode, this folded His-His_73_-ligated conformer mediates the electrocatalytic reduction of H_2_O_2_ (Figure 5), according to the same catalytic mechanism discussed above for urea-unfolded species (Equation (5)) [15,38,102].

This further confirms that the catalytic activity of cytc requires that at least one of the axial coordination positions of the ferrous heme is free, or occupied by a weak ligand, to bind the substrate molecule. Most interestingly, as the His/Met ligated form prevailing below pH 5.5 is catalytically inactive, the pseudo-peroxidase activity of the K72A/K73H/K79A mutant can be turned on and off at will, by simply adjusting the pH within a small range around the physiological conditions [15]. The Michaelis–Menten equation yielded *j_max_* and *K_M_* values of 0.5 μA cm^−2^ and 0.95 μM for the folded His-His_73_-ligated conformer (Table 2), respectively [15].

Both the electrode-immobilized folded His-His_73_ and His/Met conformers of the K72A/K73H/K79A mutant experience urea-induced unfolding [38]. The former species undergoes a conformational change above 5 M urea, involving the replacement of His73 by another endogenous His (His26 or 33) as an axial iron ligand [38]. The axial Met80 of the latter form is proposed to be replaced by the engineered His73 at low urea concentrations, which in turn is replaced by another His ligand at larger denaturant concentrations [38]. Both unfolded bis-His ligated conformers observed at high urea concentrations feature nearly coincident reduction thermodynamics, rate of heterogeneous ET, and catalytic efficiency, indicating that they correspond to the same protein form [38]. The unfolding-induced increased solvent accessibility of the heme in these systems results in a much higher catalytic efficiency compared to the folded His-His_73_-ligated species observed at pH 7.4 in the absence of denaturant (Table 2). Their lower *K_M_* is consistent with the different His axial ligand, which apparently binds to the heme center more weakly than His73, increasing the heme affinity for H_2_O_2_ [38]. Likewise, the unfolded bis-His ligated conformers of the K72A/K73H/K79A mutant feature much lower *K_M_* values than the urea-unfolded wt cytc and KtoA variants (Table 2), which indicates the easier access to the heme by H_2_O_2_ and suggests slightly different heme environments [38]. Most importantly, the observed decrease in K_M_ results in a much higher catalytic efficiency.

### 3.2. Adsorption-Induced Unfolding

Immobilization on a hydrophobic SAM of decane-1-thiol (1-DT) induces the unfolding of wt and KtoA mutated cytc, resulting in an equilibrium between low-potential (LP) and high-potential (HP) conformers [103,104]. Surface-enhanced resonance Raman (SERR) spectra demonstrated that the LP and HP species contain a low-spin (LS) six-coordinated His-His and a high-spin (HS) five-coordinated His/− heme, respectively [103]. This assignment is supported by the corresponding *E*°′ values, since the reduction potential of the LP conformer resembles those of the His-His ligated urea-unfolded cytc [101,102,113,115,136] and diheme cytochrome *c* (DHC) from *Shewanella baltica* OS155 [142], whereas the *E*°′ of the HP conformer is similar than that of immobilized myoglobin, featuring a five-coordinated high-spin His/− ligated heme [143]. Moreover, the LS six-coordinated His-His ligation prevails in the oxidized state, whereas the HS five-coordinated His/− heme prevails in the reduced form [103,104].

Gold electrodes modified with a SAM of decane-1-thiol (1-DT) were used to study the effect of hydrophobic immobilization on the redox behavior of the adduct formed by wt and KtoA cytc with cardiolipin (CL) [103,104], a negatively charged glycerophospholipid found in the inner mitochondrial membrane (IMM) [48,57,62,68,75,77,93,106,107]. Binding of cytc to CL in vivo blocks the mitochondrial electron transfer chain and imparts the protein with a significant (lipo)peroxidase activity [48,57,68,75,77,93,97,100,106,107]. CL peroxidation catalyzed by CL-bound cytc increases the permeability of the mitochondrial membrane, allowing cytc ejection into the cytosol, where it promotes the apoptosis cascade, upon forming a multimeric complex (apoptosome) with Apaf-1 (Apoptotic protease activating factor 1), ATP, and pro-caspase-9 [48,57,68,75,77,93,106,107]. The interaction between cytc and CL has been widely investigated in solution, revealing that formation of the cytc-CL adduct induces a relevant conformational change, which includes swapping of the axial methionine ligand [48,57,62,68,75,77,93,95,96,97,100,106,107,144]. 

Although not strictly relevant for biosensing, hydrophobic immobilization of the cytc-CL adduct provides important bio-physical information, since it mimics the motional restriction experienced by cytc upon binding to CL at IMM and it conceivably would more closely reproduce the in vivo behavior than the studies carried out in solution [103,104]. Moreover, the extended and smoothed surface of the SAM of 1-DT is more similar to the IMM surface compared to a small, soft, and approximately spherical liposome in solution [103,104], since the protein density on the phospholipidic membrane and its curvature heavily affect the cytc–CL interaction [68,92].

Adsorption of cytc–CL adducts on the hydrophobic SAM of decane-1-thiol results in the equilibrium between the same low-potential (LP) low-spin six-coordinated His-His and high-potential (HP) high-spin five-coordinated His/− conformers observed without CL, prevailing in the oxidized and reduced states, respectively [103,104]. Interestingly, CL binding makes the conversion between the LP and HP conformers faster. This behavior is similar to that observed upon the cytc binding to liposomes formed by 100% CL or by a combination of 1,1′,1,2′-tetraoleyolcardiolipin (TOCL, 20%) and 1,2-deoleyol-snglycero-3-phosphocholine (DOPC, 80%) using different spectroscopic techniques [95,109].

Upon increasing the concentration of O_2_ (by increasing the time of exposure of the electrochemical cell, initially under argon, to air) [103] and H_2_O_2_ [104], the current of the cathodic peak of the LP His-His species invariably increases, with almost no change in peak potential, as observed for the urea-unfolded wt and KtoA cytc immobilized on anionic and hydrophilic SAMs [101,102]. This demonstrates that the ferrous LP conformer can mediate the electrocatalytic reduction of O_2_ and H_2_O_2_, according to the same catalytic mechanisms discussed above for urea-unfolded species (Equations (4) and (5), respectively), which assume the dissociation of one of the axial His from the iron(II) center. Unfortunately, the cathodic peak of the HP conformer is barely detectable in the applied experimental conditions and cannot be considered in the analysis of the observed electrocatalytic activity [103,104]. The catalytic activity of free and CL-bound cytc immobilized on the SAM of decane-1-thiol (1-DT) for the reduction of H_2_O_2_ can be estimated using the usual Michaelis–Menten approach, yielding *K_M_* and *j_max_* values comparable to those of urea-unfolded wt and KtoA cytc immobilized on negatively charged SAMs (Table 2). The CL adducts invariably feature a slightly higher electrocatalytic efficiency, due to an increased *K_M_* and *j_max_* [104]. Hence, in the presence of CL, a slight decrease in the accessibility of the catalytic center is observed, whereas a larger *j_max_* should reflect an enhancement of *k_cat_*.

Since high concentrations of O_2_^−^ can trigger cell death, and spontaneous bursts of superoxide generation in mitochondria are early signals initiating oxidative stress-related apoptosis [103]; the ability to catalytically reduce O_2_ to O_2_^−^ conferred on the cytc/CL adducts by immobilization on 1-DT can be physiologically relevant, possibly suggesting a direct involvement of cytc and O_2_ in superoxide ion generation during pre-apoptotic processes in mitochondria [103].

## 4. Combining Point Mutations with Unfolding

The M80A and M80A/Y67A variants of yeast cytc were immobilized on a MUA/MU modified Au electrode and denatured with urea [44]. SERR spectra show that the two immobilized (folded) mutants contain a low-spin six-coordinate heme, the axial coordination positions of which are occupied by the proximal His residue and a hydroxide ion [44], as observed in solution [9,37,122,123,127]. The same heme axial coordination is maintained upon urea-induced unfolding at neutral pH, although an increased accessibility of the heme pocket to the solvent is observed [44]. Most importantly, the two immobilized mutants can catalyze the reduction of H_2_O_2_ (pseudo-peroxidase activity) and NO_2_^−^ (nitrite-reductase activity), in both folded and urea-unfolded states [44]. As observed for other mutant/surface combinations, the electrocatalytic activity towards H_2_O_2_ was irreversibly lost for substrate concentrations above 7 μM, while at lower concentrations the adduct was found to be both reusable and stable, which are crucial requirements for operation in real life scenarios. The *K_M_* values for the pseudo-peroxidase activity were 1.38 and 5.36 μM for the urea-unfolded M80A and M80A/Y67A, respectively [44], which are both lower than the corresponding values for the folded mutants in the absence of urea (Table 2). This finding was ascribed to an increased kinetic instability of the Fe(III)-hydroperoxide complex that should be formed during the catalytic cycle, which is also responsible for the larger catalytic activity of the unfolded immobilized mutants compared to the folded species, as indicated by their larger *j_max_/K_M_* ratios [44]. On the contrary, the opposite effects of urea unfolding on the *j_max_* of the two mutants could not be unambiguously explained.

Moreover, with respect to the nitrite-reductase activity observed with NO_2_^−^ concentrations in the 1–6 μM range, urea-induced unfolding yields a decrease in *K_M_* if compared to the folded species (Table 1). This finding can again be explained by invoking the higher accessibility of the heme iron to exogenous substrates [92]. The effect is more pronounced for M80A than for M80A/Y67A, with the latter displaying much lower kinetic instability, most likely due to a more hydrophobic catalytic pocket (following the Tyr-to-Ala mutation) that impacts on the accessibility to the ionic substrate [44].

Upon immobilization on gold electrodes modified with a SAM of decane-1-thiol, the M80A and M80A/Y67A yeast cytc mutants and their CL-adducts show the same behavior as the wt protein and its triple KtoA in the same conditions [39]. In particular, the same low potential (LP) low-spin six-coordinated His/His and high potential (HP) high-spin five-coordinated His/− conformers are observed, which are more stable in the oxidized and reduced states, respectively [39]. Hence, immobilization on the hydrophobic surface invariably results in the release of the OH^−^ ion, which replaces the native Met80 as heme axial ligand in both mutants, either in solution or electrostatically bound to a negatively charged SAM of MUA/MU [44]. Interestingly, the interconversion of the LS 6c His/His (LP) species to the HS 5c His/− (HP) conformer is favored by increasing temperature [39]. This effect was not observed for the wt protein and its triple KtoA mutant [103,104]. Overall, the above results indicate that (i) the heme axial ligation in free or CL-bound M80A and M80/Y67A immobilized on a hydrophobic surface is different from that observed in solution and upon electrostatic immobilization on a MUA/MU SAM, and (ii) the coordination of the heme center in both mutants is not appreciably affected by CL binding [39].

In the presence of micromolar concentrations of H_2_O_2_ and NO_2_^−^, the M80A and M80A/Y67A mutants and their CL-adducts immobilized on decane-1-thiol-modified Au electrodes show good pseudo-peroxidase and nitrite-reductase activities, which are observed up to H_2_O_2_ and NO_2_^−^ concentrations of about 5 μM and 1.75 μM, respectively [39]. Below these concentrations, elimination of substrates restores the voltammetric signal of the protein, while catalytic currents could be re-obtained upon addition of hydrogen peroxide and sodium nitrite. Hence, under the applied conditions, the protein layer is stable and re-usable.

For both substrates, the ferrous His/His-ligated forms are the catalytically active species, indicating that their catalytic reduction by M80A and M80A/Y67A immobilized on 1-DT requires detachment of the 6th His ligand from the Fe(II) heme [39]. Therefore, the catalytic mechanism for H_2_O_2_ reduction is the same as suggested for wt cytc and its triple KtoA variant subjected to the same immobilization [90], while the following mechanism is proposed for NO_2_^−^ reduction [39]:

cytc-Fe(III)-His + e^−^ → cytc-Fe(II) − − − His  cytc-Fe(II) − − − His + NO_2_^−^ + H_2_O →cytFe(III)-His + NO + 2OH^−^ (6)

The usual approach based on the Michaelis–Menten equation expressed in terms of current density is used to estimate the catalytic activity of free and CL-bound M80A and M80A/Y67A immobilized on the SAM of decane-1-thiol (1-DT) for H_2_O_2_ and NO_2_^−^ reduction. The resulting *K_M_* for both substrates are sensibly larger than those for other immobilized heme proteins (Table 1 and Table 2) and the *K_M_* for H_2_O_2_ are comparable to those of wt cyt and its triple KtoA mutant (Table 2).

**Figure 6:**
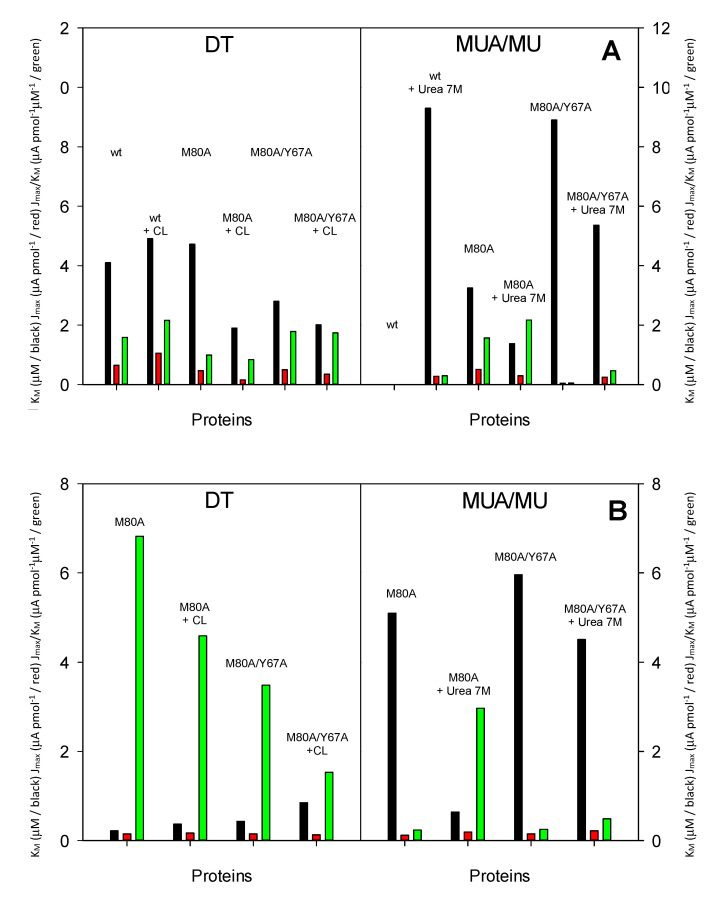
Histograms depicting the *K_M_* (μM, black bar), *J_max_* (μA pmol^−1^, red bar) and *J_max_*/*K_M_* × 10 (μA pmol^−1^ μM^−1^, green bar) values for the reductive electrocatalysis of (**A**) H_2_O_2_ and (**B**) NO_2_^−^, carried out by wt yeast iso-1 cytochrome *c* and its M80A and M80A/Y67A variants immobilized on a gold electrode functionalized with a hydrophobic SAM of decane-1-thiol (DT) and a negatively charged SAM of MUA/MU, along with the corresponding adducts with cardiolipin (this work) and the same species in 7M urea immobilized under the same conditions. Figure (**A** left frame): 1, wt; 2, wt + CL, 3, M80A; 4, M80A + CL; 5, M80A/Y67A; 6, M80A/Y67A + CL. Figure (**A** right frame): 1, wt; 2, wt + Urea 7M, 3, M80A; 4, M80A + Urea 7M; 5, M80A/Y67A; 6, M80A/Y67A + Urea 7M. Figure (**B** left frame): 1, M80A; 2, M80A + CL; 3, M80A/Y67A; 4, M80A/Y67A + CL. Figure (**B** right frame): 1, M80A; 2, M80A + Urea 7M; 3, M80A/Y67A; 4, M80A/Y67A + Urea 7M. Reprinted with permission from ref. [39]. Copyright 2020 Elsevier.

The effects on the electrocatalytic activity of the mutation(s) within the heme site and CL binding are reaction-specific (Figure 6). For hydrogen peroxide reduction, the different hydrogen bonding network within the heme crevice disfavors access of the substrate to the heme iron in the M80A mutant and facilitates it in the M80A/Y67A species, resulting in a larger and a lower *K_M_* compared to the wild type protein (Table 2) [39]. Likewise, the different heme environment is responsible for the opposite effect being exerted by CL binding on the *K_M_* of wt cytc and the two mutants (Figure 6). It is important to stress that the observed nitrite-reductase activity results from the M80A mutation, since the wt protein is catalytically inactive [39] (Figure 6 and Table 1). Contrary to the pseudo-peroxidase reaction, the *K_M_* value for nitrite reduction increases upon addition of the Y67A mutation and/or formation of the CL-adducts (Figure 6 and Table 1), possibly reflecting the enhanced hydrophobicity of the heme crevice, which would hamper the access of the negatively charged NO_2_^−^ to the metal center [39]. 

Comparison of the data reported in Table 1 and Table 2 unequivocally demonstrates that the electrocatalytic ability of wt cytc and its M80A and M80A/Y67A mutants is strongly affected by the nature of the SAM used for protein immobilization. In particular, the absence of pseudo-peroxidase activity for wt yeast cytc on MUA/MU SAM [102] is related to the persistence of the Met-Fe(II) axial bond, which hampers substrate binding, whereas the weaker and more labile His-Fe(II) bond observed upon immobilization on DT allows for H_2_O_2_ binding and turnover [39]. On the other hand, the different mutation-induced changes in the catalytic activity observed for the same species upon immobilization on the two different SAMs show that the influence of protein-SAM interactions on protein conformation extends to the H-bonding network, polarity, solvation, and electrostatics of the heme environment [39].

## 5. Conclusions

We have shown that two main routes can be followed to turn cytochrome *c* into a redox enzyme to be immobilized on an electrode for biosensing applications. The first is the design and production of ad hoc designed mutants. To this end, the most widely explored mutation has been the substitution of the axial heme iron ligand Met80 into a non-coordinating alanine to yield a free position in the iron coordination sphere [8,9,37]. Other mutations have been explored, mainly aimed at altering the hydrogen bond network in the heme pocket (Tyr67) [9] or introducing a His residue on the protein surface (Lys73) that can serve as weak axial ligand in a pH-switchable, low potential conformer [15,38].

The second strategy is to unfold cytc. In this respect, urea-induced unfolding has been often preferred, generating a His-His conformer possessing catalytic properties absent in the folded species [101,102]. Cytc can also be turned into a redox enzyme upon immobilization on a hydrophobic SAM [103,104]. Moreover, site directed mutagenesis and chemical- or adsorption-induced unfolding can also be used in synergy [39,44]. Dioxygen, hydrogen peroxide, and nitrite anions are the three main substrates that have been successfully investigated. The catalytic activity of the novel, non-native species have been described using the Michaelis–Menten equation, expressed in terms of current density [8,9,15,38,39,44,101,102,104]. The combination of the main structural features of cytc (e.g., a covalently bound heme, stability over a large range of conditions) with the enzymatic properties introduced ad hoc make this protein a strong candidate for biosensing applications, provided the issues related to its lower catalytic activity and selectivity compared to redox enzymes can be effectively solved.

## Figures and Tables

**Table 1:** Kinetic parameters for the electrocatalytic reduction of NO_2_^−^ carried out by wt yeast iso-1 cytochrome *c* (ycc) and some of its variants covalently bound to a bare polycrystalline gold electrode (M80A/N62C/C102T) or adsorbed on a polycrystalline gold electrode coated with a SAM of MUA/MU or 1-DT (M80A and M80A/Y67A). For the solution composition, experimental conditions, and uncertainties associated with the reported values, the reader is referred to the original literature.

	*K_M_* (μM)	*J_max_* (μA cm^−2^)	*J_max_/K_M_* (μA cm^−2^ μM^−1^)	Ref.
Protein covalently bound to a bare Au electrode				
M80A/N62C/C102T ^a^	5.1	2.14	0.42	[8]
Protein adsorbed on a Au electrode surface-modified with a MUA/MU SAM				
wt ycc folded	-	-	-	[44]
wt ycc unfolded (7 M urea)	-	-	-	[44]
M80A ycc unfolded (7 M urea)	0.64	3.47	5.42	[44]
M80A/Y67A ycc folded	5.96	2.78	0.47	[44]
M80A/Y67A ycc unfolded (7 M urea)	4.51	3.95	0.88	[44]
Protein adsorbed on a Au electrode surface-modified with a 1-DT SAM				
wt ycc	-	-	-	[39]
wt ycc + CL	-	-	-	[39]
M80A ycc	0.22	1.20 ^b^	5.46 ^b^	[39]
M80A ycc +CL	0.37	1.50 ^b^	4.04 ^b^	[39]
M80A/Y67A ycc	0.43	1.42 ^b^	3.30 ^b^	[39]
M80A/Y67A ycc + CL	0.85	1.35 ^b^	1.59 ^b^	[39]

^a^ These kinetic parameters correspond to those for the electrocatalytic reduction of NO_2_^−^ by the folded M80A variant, since covalent binding of the M80A/N62C/C102T variant to a bare polycrystalline gold electrode does not induce protein unfolding. ^b^ Calculated by multiplying the data reported in Table 3 of ref. [39] by the corresponding electrode surface coverages Γ_0_.

**Table 2:** Kinetic parameters for the electrocatalytic reduction of H_2_O_2_ carried out by wt beef heart cytochrome *c* (bcc), wt yeast iso-1 cytochrome *c* (ycc) and its K79A, K73A, K72A/K73A, K72A/K73A/K79A, K72A/K73H/K79A, M80A, M80A/Y67A, and M80A/Y67H variants adsorbed on a polycrystalline gold electrode coated with a SAM of MUA/MU or 1-DT. For the solution composition, experimental conditions, and uncertainties associated with the reported values, the reader is referred to the original literature.

	*K_M_* (μM)	*J_max_* (μA cm^−2^)	*J_max_/K_M_* (μA cm^−2^ μM^−1^)	Ref.
Protein adsorbed on a Au electrode surface-modified with a MUA/MU SAM				
wt ycc	-	-	-	
wt ycc unfolded (8 M urea)	9.3	5.18	0.56	[102]
K79A ycc unfolded (8 M urea)	2.0	5.50	2.75	[102]
K73A ycc unfolded (8 M urea)	18.8	2.68	0.14	[102]
K72A/K73A ycc unfolded (8 M urea)	18.3	5.53	0.30	[102]
K72A/K73A/K79A ycc unfolded (8 M urea)	11.5	2.34	0.20	[102]
K72A/H73H/K79A ycc folded (0 M urea), pH 7.4	0.95	0.5	0.5	[38]
K72A/H73H/K79A ycc unfolded (4 M urea), pH 7.4	0.96	4.0	4.2	[38]
K72A/H73H/K79A ycc unfolded (8 M urea), pH 7.4	0.60	2.7	4.5	[38]
K72A/H73H/K79A ycc unfolded (8 M urea), pH 5	0.59	2.8	4.7	[38]
M80A ycc	3.25	9.26	2.85	[9]
M80A ycc unfolded (7 M urea)	1.38	5.49	3.98	[44]
M80A/Y67A ycc	8.90	0.94	0.11	[9]
M80A/Y67A ycc unfolded (7 M urea)	5.36	4.52	0.84	[44]
M80A/Y67H ycc	3.7	2.91	1.27	[9]
Protein adsorbed on a Au electrode surface-modified with a 1-DT SAM				
wt bcc	3.4	12.4	3.56	[104]
wt bcc + CL	4.1	17.3	4.22	[104]
wt ycc	4.1	11.2	2.73	[104]
ycc wt + CL	4.9	16.5	3.37	[104]
K72A/K73A/K79A ycc	5.5	10.8	1.96	[104]
K72A/K73A/K79A + CL	6.2	15.1	2.44	[104]
M80A ycc	4.72	3.76 ^a^	0.80 ^a^	[39]
M80A/Y67A ycc	2.80	4.73 ^a^	1.69 ^a^	[39]
M80A ycc +CL	1.90	1.41 ^a^	0.74 ^a^	[39]
M80A/Y67A ycc + CL	2.01	3.64 ^a^	1.81 ^a^	[39]

^a^ Calculated by multiplying the data reported in Table 3 of ref. [39] by the corresponding electrode surface coverages Γ_0._

**Table 3:** Kinetic parameters for the electrocatalytic reduction of O_2_ carried out by urea-unfolded bis-His form of yeast iso-1 cytochrome *c* (ycc) adsorbed on a polycrystalline gold electrode coated with a SAM of MUA/MU or 4-MP. For the solution composition, experimental conditions, and uncertainties associated with the reported values, the reader is referred to the original literature.

	*K_M_* (Pa)	*J_max_* (μA cm^−2^)	Ref.
wt ycc adsorbed on MUA/MU	2.39 × 10^4^	4.75	[101]
wt ycc adsorbed on 4-MP	1.76 × 10^4^	2.99	[101]

## Data Availability

No new data were created or analyzed in this study. Data sharing is not applicable to this article.
